# A phase I and pharmacokinetic study of intraperitoneal topotecan

**DOI:** 10.1054/bjoc.2001.2161

**Published:** 2001-11

**Authors:** L S Hofstra, A M E Bos, E G E de Vries, A G J van der Zee, J H Beijnen, H Rosing, N H Mulder, J G Aalders, P H B Willemse

**Affiliations:** Department of Medical, Gynaecologic Oncology, University Hospital Groningen, PO Box 30.001, Groningen, RB, 9700, The Netherlands; Department of Pharmacy and Pharmacology, Slotervaart Hospital, Louwesweg 6, Amsterdam, EC, 1066, The Netherlands

**Keywords:** intraperitoneal, topotecan, ovarian cancer, pharmacokinetics, phase I

## Abstract

*Purpose*: To evaluate the feasibility and pharmacology of intraperitoneal (IP) topotecan. *Patients and methods*: Fifteen patients with recurrent ovarian cancer in a phase I trial were treated with escalating IP topotecan doses (5–30 mg/m^2^) for pharmacokinetic analysis. *Results*: Dose limiting toxicity (DLT) was acute hypotension, chills and fever at the 30 mg/m^2^ dose level. Haematological toxicity and abdominal pain were mild for all dose levels studied. *Pharmacokinetics*: Peak plasma levels of total topotecan were reached at 2.7 ± 1.1 h after IP instillation. The apparent V _ss_ was 69.9 ± 25.4 L/m^2^, plasma clearance 13.4 ± 2.5 L/h/m^2^ and plasma T1/2 3.7 ± 1.3 h. The plasma AUC was correlated with the dose (R = 0.95, *P* < 0.01). The plasma AUC ratio of lactone versus total topotecan (lactone + carboxy-forms) increased with the dose from 16% to 55%, (R = 0.84, *P* < 0.01). Peritoneal total topotecan was cleared from the peritoneal cavity at 0.4 ± 0.3 L/h.m^2^ with a T1/2 = 2.7 ± 1.7 h. The mean peritoneal/plasma AUC ratio for total topotecan was 54 ± 34. *Conclusion*: A substantial dose of topotecan can be delivered by the IP route, achieving cytotoxic plasma levels of topotecan, with acceptable toxicity. The recommended dose for further phase II trials is 20 mg/m^2^ IP, which enables combination with active doses of other cytotoxic drugs, in view of its limited myelotoxicity when given by this route. © 2001 Cancer Research Campaign http://www.bjcancer.com


*British Journal of Cancer* (2001) **85**(11), 1627–1633
© 2001 Cancer Research Campaign
doi: 10.1054/ bjoc.2001.2161, available online at http://www.idealibrary.com on IDEAL
http://www.bjcancer.com

### A phase I and pharmacokinetic study of intraperitoneal topotecan

**LS Hofstra[1], AME Bos[1], EGE de Vries[1], AGJ van der Zee[2], JH Beijnen[3], H Rosing[3], NH Mulder[1], JG Aalders[2] and PHB Willemse[1]**

Department of [1]Medical and [2]Gynaecologic Oncology, University Hospital Groningen, PO Box 30.001, 9700 RB Groningen, The Netherlands; and [3]Department of Pharmacy and Pharmacology, Slotervaart Hospital, Louwesweg 6, 1066 EC Amsterdam, The Netherlands

**Summary** *Purpose*: To evaluate the feasibility and pharmacology of intraperitoneal (IP) topotecan. *Patients and methods*: Fifteen patients with recurrent ovarian cancer in a phase I trial were treated with escalating IP topotecan doses (5–30 mg/m$^2$) for pharmacokinetic analysis. *Results*: Dose limiting toxicity (DLT) was acute hypotension, chills and fever at the 30 mg/m$^2$ dose level. Haematological toxicity and abdominal pain were mild for all dose levels studied. *Pharmacokinetics*: Peak plasma levels of total topotecan were reached at 2.7 ± 1.1 h after IP instillation. The apparent $V_{ss}$ was 69.9 ± 25.4 L/m$^2$, plasma clearance 13.4 ± 2.5 L/h/m$^2$ and plasma T1/2 3.7 ± 1.3 h. The plasma AUC was correlated with the dose (R = 0.95, $P < 0.01$). The plasma AUC ratio of lactone versus total topotecan (lactone + carboxy-forms) increased with the dose from 16% to 55%, (R = 0.84, $P < 0.01$). Peritoneal total topotecan was cleared from the peritoneal cavity at 0.4 ± 0.3 L/h.m$^2$ with a T1/2 = 2.7 ± 1.7 h. The mean peritoneal/plasma AUC ratio for total topotecan was 54 ± 34. *Conclusion*: A substantial dose of topotecan can be delivered by the IP route, achieving cytotoxic plasma levels of topotecan, with acceptable toxicity. The recommended dose for further phase II trials is 20 mg/m$^2$ IP, which enables combination with active doses of other cytotoxic drugs, in view of its limited myelotoxicity when given by this route. © 2001 Cancer Research Campaign http://www.bjcancer.com

**Keywords**: intraperitoneal; topotecan; ovarian cancer; pharmacokinetics; phase I

Topotecan (Hycamtin), a semisynthetic water-soluble derivate of camptothecin, is a potent inhibitor of DNA topoisomerase I in vitro (Kingsbury et al, 1991) and has demonstrated promising anti-tumour activity in a wide variety of tumours, including ovarian cancer and small cell lung cancer (Ten Bokkel Huinink et al, 1997; Ardizzonni et al, 1997). A recent study showed that topotecan has efficacy at least equivalent to paclitaxel manifested by a higher response rate and longer time to progression in patients with recurrent epithelial ovarian cancer and it has shown cytotoxic activity in patients refractory to platinum and paclitaxel (Ten Bokkel Huinink et al, 1997). In patients who failed platinum based chemotherapy, response rates ranged from 13% to 25% with a duration of 22–32 weeks (Neijt et al, 1991; Kudelka et al, 1996; Ten Bokkel Huinink et al, 1997). Topotecan binds with the topoisomerase I-DNA complex and interferes with the process of DNA breakage and resealing, resulting in DNA breaks, fragmentation and cell death (Hertzberg et al, 1989; Hsiang et al, 1989). To date, activity has been observed primarily with continuous and frequent dosing schedules, particularly when topotecan is given as a 1.5 mg/m$^2$/day infusion on 5 consecutive days every 21 days, with myelotoxicity as the dose limiting factor (Kudelka et al, 1996; Ten Bokkel Huinink et al, 1997). Under physiological conditions the active lactone moiety of topotecan undergoes a rapid and reversible pH-dependent conversion into a carboxylated open-ring form, with less topoisomerase I inhibiting activity. At pH 7.4 the open-ring form predominates at equilibrium and topotecan is stable in infusion fluids at pH < 4.0, but unstable in plasma (Rowinsky et al, 1992; Verweij et al, 1993; Herben et al, 1996).

Direct intraperitoneal (IP) installation of some cytotoxic agents offers the potential of exposing the IP tumour to high concentrations with less of the usual systemic side effects (Markman et al, 1992; Markman, 1998a). Randomized studies have shown less toxicity and an increased disease free survival in the group treated with IP chemotherapy (Alberts et al, 1996; Markman et al, 1998b; Hofstra et al, 2000). A study on the feasibility of a triple drug schedule with cisplatin, paclitaxel and topotecan has shown that full IV doses of these drugs can not be reached due to severe myelotoxicity (Herben et al, 1999). We wanted to study if IP instillation of full doses of topotecan would be possible without prohibitive myelotoxicity, with the aim to combine this drug with standard doses of the two other compounds in a subsequent study. Therefore, the aim of this study was to investigate the safety and pharmacokinetic properties of IP topotecan in patients with recurrent ovarian cancer.

*Received 27 November 2000*
*Revised 2 August 2001*
*Accepted 17 September 2001*

*Correspondence to:* PHB Willemse

### PATIENTS AND METHODS

### Patients

Eligible patients were between 18 and 75 years of age with advanced recurrent ovarian cancer and with a life expectancy of > 12 weeks. In the absence of histological evidence of disease progression, patients could be entered into this trial on the basis of repeated elevated CA125 levels. Patients had received at least one prior treatment with platinum- and paclitaxel containing chemotherapy, but no topotecan or other topo-isomerase inhibitors. All patients had a performance status of 0–2, according to WHO criteria, normal blood counts (leucocytes $3.0 \times 10^9$/L, platelets 100

× $10^9$/L) and adequate renal-(serum creatinine 1.5 × upper normal limit) or liver function (bilirubin 1.5 × upper normal limit). Exclusion criteria included patients with borderline ovarian tumours or germ cell tumours, complete bowel obstruction, a history of atrial or ventricular arrhythmias, congestive heart failure, or documented myocardial infarction within the previous 6 months. Patients with active infection or other serious medical conditions were also excluded. Finally, patients with peritoneal adhesions which preclude homogenous distribution of peritoneal fluid were excluded. The study was approved by the local Ethical Committee. All patients gave written informed consent.

### Treatment plan

All patients had a surgically implanted peritoneal-access-port (PAP) catheter (Arts et al, 1998). Before administration of IP topotecan, fluid distribution was controlled by the instillation of $^{99}$Tc-colloid and an even distribution over all four quadrants was obligatory for study entry. (Levenback et al, 1994). Formulated topotecan, dissolved in 1 L of normal saline (pH = 4) was infused over 1 h into the abdominal cavity, after previous infusion of 1 L of normal saline at 37˚C on day 1. The projected dose steps of topotecan were 5–10–15–20–30 mg/m$^2$/day IP in 3 patients on each dose level.

Cycles were repeated every 3 weeks to a maximum of 8. Ondansetron 8 mg was administered IV 1 h prior to topotecan infusion. Standard slow-release morphine 20 mg orally and rectal diazepam 10 mg were administered once before topotecan infusion in order to minimize acute abdominal discomfort to keep the patient comfortable during the period of IP infusion. No steroids were given before or after topotecan. In case of inadequate bone marrow recovery on day 21 (leucocytes <3.0 × $10^9$/L or platelets <100 × $10^9$), administration was delayed for 1 week. Patients went off study in case of tumour progression, topotecan hypersensitivity, or after completing 8 treatment cycles. Non-haematologic toxicity grade III or higher, infection or bleeding due to haematologic toxicity or haematological toxicity grade IV led to dose reduction to the nearest level and repeated haematological toxicity grade IV to discontinuation of treatment. For the determination of dose-limiting toxicity and the MTD, grade IV haematologic and grade III non-haematologic toxicity were considered as dose-limiting for this study.

### Assessment

At baseline and before each cycle of therapy, a physical examination was performed, with a complete blood count, serum CA125 (IMX Ca-125, Abbott Diagnostics, Chicago, IL) and blood chemical measurements. On day 8 and 15 of each cycle a complete blood count was performed. All initially positive radiology (CT-scan, ultrasound) examinations were repeated after completion of therapy. Toxicities were recorded according to the WHO grading system. A complete response was defined as no clinical evidence of disease including a normalized CA125 level.

### METHODS

### Topotecan pharmacokinetics

Pharmacokinetic sampling was performed on day 1 of the first treatment cycle. Serum and IP fluid samples were collected at 0, 1, 2, 4, 6, 10 and 24 h after the start of IP topotecan, which was administered at 37˚C over 1 h. Plasma was withdrawn by IV sampling and IP-fluid via the PAP-catheter and both were collected in heparinized tubes on ice. Before the collection of each IP sample, 2 mL of peritoneal fluid was discarded for potential topotecan-residue in the catheter (internal volume about 0.5 mL). All samples were centrifuged immediately after sampling at 3000 g for 5 min at 4˚C. To prevent conversion of the lactone form to the open form, a volume of 1.0 ml methanol at –20˚C was added to 0.5 mL of the separated plasma. The sample was then centrifuged for 5 min and the supernatant was transferred to a clean tube. All samples were stored at –80˚C until analysis. Plasma and peritoneal fluid levels of topotecan were determined using a validated high-performance liquid chromatography (HPLC) method with UV detection as previously reported (Rosing et al, 1995; Herben, 1996). The plasma concentration curves were fitted by using the Kinfit computer program (MW\Pharm, Medi\Ware BV, Groningen, The Netherlands) (Proost and Meijer, 1992). Kinetic parameters were calculated using standard equations. The total Area Under the Concentration-time curve (AUC), was calculated by the linear trapezoidal method. For the calculation of the other parameters however, the fitted AUC curve according to the computed exponential model was applied.

### RESULTS

Patient characteristics are summarized in Table 1. A total of 75 cycles of IP topotecan were administered to 15 patients enrolled in the study. Dose steps and dose limiting haematologic toxicity are displayed in Table 2. In one patient at 15 mg/m$^2$ and one at 30 mg/m$^2$ the dose level was reduced for toxicity as described below.

**Table 1** Patient characteristics (*n* = 15)

| | |
|---|---|
| Number of patients | 15 |
| Age (years) | |
| median | 55 |
| range | 41–68 |
| WHO Performance status | |
| 0 | 9 |
| 1 | 5 |
| 2 | 1 |
| Prior chemotherapy regimens | |
| IP paclitaxel with IV carboplatin/cyclophosphamide | 15 |
| Second line treatment with | |
| Oral etoposide | 2 |
| Oral L-PAM | 2 |

**Table 2** Topotecan dose level, number of cycles and worst haematologic toxicity per dose level

| opotecan IP dose (mg/m$^2$) | No. of patients | No. of cycles | Toxicity: WBC ($10^9$/L) Grade III | Grade IV | Platelets ($10^9$/L) Grade III | Grade IV |
|---|---|---|---|---|---|---|
| 5 | 3 | 10 | 0 | 0 | 0 | 0 |
| 10 | 3 | 13 | 0 | 0 | 0 | 0 |
| 15 | 3 | 21 | 1 | 1 | 1 | 0 |
| 20 | 3 | 22 | 2 | 0 | 1 | 0 |
| 30 | 3 | 8 | 2 | 0 | 0 | 0 |
| Total | 15 | 74 | 5 | 1 | 2 | 0 |

 *© 2001 Cancer Research Campaign*

Only mild to moderate (grade I-III) haematological toxicity was encountered starting from the 15 mg/m² dose level. At 15 mg/m² one patient experienced a grade IV leukopenia and grade III thrombocytopenia during the first treatment cycle; subsequent treatment with 10 mg/m² did not give further myelosuppression. Four of the six patients treated at higher doses had grade III leukopenia and one patient had a grade III thrombocytopenia (Table 2). The duration of myelosuppression was short (< 7 days) and non-cumulative and haematological toxicity resulted in dose reduction in two patients but no delay of the next cycle. One admission on day 9–11 for grade IV neutropenic fever was recorded at the 30 mg/m² dose level. No bleeding episodes occurred.

### Non-haematological toxicity

Dose limiting toxicity (DLT) was encountered at the 30 mg/m² dose level (Table 3). DLT was an acute reaction in one patient immediately after infusion of the full dose of topotecan comprising severe hypotension, fever (39.7˚C) and chills without an infectious focus. Rapid treatment with volume expanders and IV antihistamines resulted in a full recovery within hours without sequelae. This patient continued with IV topotecan 1.5 mg/m²/day over 5 days every 21 days without further problems. She had had no prior exposure to topo-I inhibitors, and had previously been treated with IP paclitaxel without local problems.

Nausea and/or vomiting grade I-II was recorded in 12 patients. Nausea and vomiting was grade III in three patients at the two highest dose levels. Patients responded to standard anti-emetics and this did not result in prolonged hospitalization.

Abdominal pain grade I was observed in six patients and was not related to the topotecan dose level. All patients treated with topotecan 15 mg/m² or more developed alopecia grade II. A generalized but transient skin rash was observed in four patients starting 3–5 days following IP administration. In one of these four patients, treated at 15 mg/m², for a rash combined with fever and grade IV leukopenia and grade III thrombocytopenia the dose was reduced to 10 mg/m². Re-treatment with topotecan did not result in a reappearance of skin rashes in one of these patients. Two of the three patients treated at 30 mg/m² dose level complained of grade II headache, which responded to acetaminophen and did not need dose-reduction or prolonged hospitalization.

### Tumour responses

In these pretreated patients no complete clinical responses were observed. Seven patients had no clinical parameter and two had no detectable marker. Partial responses, defined by a > 50% decrease in repeated CA125 levels were observed in 6 of 13 biochemically evaluable patients. Stable disease for 6–8 cycles was observed in 5 of 8 clinically evaluable patients. Progressive disease, defined by the appearance of new lesions or an increase by > 50% in tumour measurements or in repeated CA125 levels was observed in six patients, three by marker and three by clinical parameters. The duration of treatment was not different for the dose levels studied.

**Table 3** Topotecan dose level, number of cycles and worst non-haematologic toxicity per dose level

| Dose (mg/m²) | | 5 | 10 | 15 | 20 | 30 |
|---|---|---|---|---|---|---|
| Number of patients | Toxicity WHO Grade | 3 | 3 | 3 | 3 | 3 |
| Nausea | II | 1 | 2 | 3 | 1 | 1 |
| | III | 0 | 0 | 0 | 2 | 1 |
| Emesis | II | 0 | 0 | 2 | 1 | 1 |
| | III | 0 | 0 | 0 | 2 | 1 |
| Rash | II | 0 | 0 | 1 | 0 | 0 |
| | III | 0 | 0 | 0 | 1 | 1 |
| Fatigue | II | 0 | 0 | 0 | 2 | 1 |
| | III | 0 | 0 | 0 | 1 | 1 |

### Topotecan IP pharmacokinetics

Peritoneal samples for pharmacokinetic analysis were available from seven patients. In the other patients, collection of peritoneal fluid from the PAP-catheter was impossible due to pericatheter fibrosis, leading to backflow valve formation and absent backflow. The intraperitoneal pharmacokinetic parameters of total topotecan are presented in Table 5A and B. The elimination phase of total topotecan (lactone plus carboxy forms) from the peritoneal cavity was best described by a mono-exponential model. The mean T1/2 for the peritoneal compartment was 2.7 ± 1.7 h. The AUC of total topotecan in peritoneal fluid was proportional with dose, as shown in Figure 1, R = 0.84, $P < 0.01$ The peritoneal to plasma AUC ratio for total topotecan was 54 ± 34. The IP lactone showed a shorter half-life of 2.3 ± 2.0 h, probably due to intraperitoneal conversion, as the lactone is unstable in a neutral pH within the abdominal cavity. As a consequence, the peritoneal lactone/total topotecan AUC ratio was 15–52%, median 29%.

### Topotecan plasma pharmacokinetics

The plasma kinetics of topotecan of 13 patients were best described by a one compartmental model. The plasma pharmacokinetic parameters of total topotecan and of lactone are presented in Table 4A and B. The plasma peak levels ($C_{max}$) of total topotecan were reached at 2.7 ± 1.1 h and of lactone 2.1 ± 0.6 h after the start of IP administration and were dose-dependent (resp. R = 0.92 and R = 0.92, for both $P < 0.01$). The mean T1/2 was 3.7 ± 1.3 h for total topotecan and 5.2 ± 2.2 h for lactone. The mean AUC, which represents the total plasma exposure time, was proportional with dose, R = 0.95, $P < 0.01$ for total topotecan

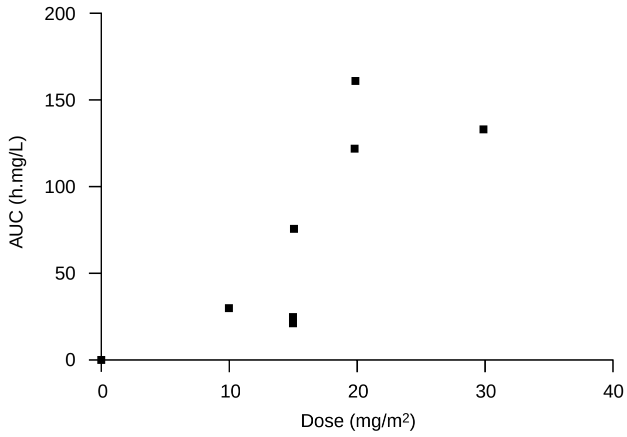

**Figure 1** AUC of peritoneal total topotecan versus dose administered in mg.h/L for individual patients, R = 0.84

© 2001 Cancer Research Campaign

**Table 4A** Plasma pharmacokinetics of total topotecan

| Patient no. | Dose (mg/m²) | Absolute dose (mg) | Body surface (m²) | AUC 0 → ∞ (h/μg/L) | CL (L/h/m²) | $V_{ss}$ (L/m²) | T1/2 (h) | MRT (h) | $k_{el}$ (x/h) | $T_{max}$ (h) | $C_{max}$ (μg/L) |
|---|---|---|---|---|---|---|---|---|---|---|---|
| 1 | 5 | 8 | 1.80 | 181 | 17.6 | 20.8 | 0.8 | 3.6 | 0.85 | 2.1 | 61.8 |
| 2 | 5 | 8 | 1.62 | 380 | 14.0 | 87.7 | 4.3 | 8.5 | 0.16 | 3.6 | 33.8 |
| 3 | 10 | 16 | 1.60 | 684 | 15.8 | 77.6 | 3.4 | 6.6 | 0.20 | 2.7 | 80.2 |
| 4 | 10 | 16 | 1.60 | 690 | 16.1 | 74.3 | 3.2 | 6.8 | 0.22 | 3.2 | 79.4 |
| 5 | 15 | 28 | 1.78 | 1153 | 13.9 | 114.6 | 5.7 | 11.9 | 0.12 | 5.4 | 75.3 |
| 6 | 15 | 28 | 1.80 | 1406 | 13.5 | 95.1 | 4.9 | 8.0 | 0.14 | 1.2 | 153.7 |
| 7 | 15 | 28 | 1.80 | 1910 | 9.1 | 49.4 | 3.8 | 6.5 | 0.18 | 1.4 | 289.4 |
| 8 | 20 | 35 | 1.75 | 1566 | 14.2 | 66.1 | 3.2 | 7.0 | 0.21 | 3.3 | 178.5 |
| 9 | 20 | 43 | 2.16 | 1434 | 12.7 | 85.6 | 4.7 | 8.1 | 0.15 | 2.6 | 169.3 |
| 10 | 20 | 36 | 1.80 | 2036 | 11.0 | 30.9 | 1.9 | 6.9 | 0.36 | 3.5 | 202.9 |
| 11 | 30 | 66 | 2.22 | 2322 | 14.7 | 74.2 | 3.5 | 6.0 | 0.20 | 1.9 | 297.2 |
| 12 | 30 | 52 | 1.75 | 2539 | 12.6 | 62.0 | 3.4 | 6.0 | 0.20 | 2.1 | 341.1 |
| 13 | 30 | 53 | 1.70 | 3784 | 9.6 | 70.7 | 5.1 | 8.6 | 0.14 | 2.4 | 331.8 |
| Mean | | | | | 13.4 | 69.9 | 3.7 | 7.3 | 0.24 | 2.7 | |
| SD | | | | | 2.5 | 25.4 | 1.3 | 1.9 | 0.19 | 1.1 | |

**Table 4B** Plasma pharmacokinetics of lactone

| Patient no. | Dose (mg/m²) | Absolute dose (mg) | Body surface (m²) | AUC 0 → ∞ (h/μg/L) | CL (L/h/m²) | $V_{ss}$ (L/m²) | T1/2 (h) | MRT (h) | $k_{el}$ (x/h) | $T_{max}$ (h) | $C_{max}$ (μg/L) |
|---|---|---|---|---|---|---|---|---|---|---|---|
| 1 | 5 | 8 | 1.80 | 81 | 52.1 | 348 | 4.6 | 7.0 | 0.15 | 1.1 | 11.0 |
| 2 | 5 | 8 | 1.62 | 66 | 78.6 | 664 | 5.9 | 9.7 | 0.12 | 2.8 | 5.3 |
| 3 | 10 | 16 | 1.60 | 107 | 98.1 | 556 | 3.9 | 6.5 | 0.18 | 1.7 | 14.0 |
| 4 | 10 | 16 | 1.60 | 131 | 83.1 | 452 | 3.7 | 7.0 | 0.18 | 2.6 | 15.3 |
| 5 | 15 | 28 | 1.78 | 185 | 81.8 | 1366 | 11.6 | 17.7 | 0.06 | 1.6 | 11.1 |
| 6 | 15 | 28 | 1.80 | 489 | 45.1 | 365 | 5.6 | 9.6 | 0.12 | 2.4 | 35.8 |
| 7 | 15 | 28 | 1.80 | 618 | 31.2 | 156 | 3.5 | 6.4 | 0.20 | 2.5 | 61.0 |
| 8 | 20 | 35 | 1.75 | 323 | 66.0 | 422 | 4.4 | 8.1 | 0.16 | 3.0 | 32.9 |
| 9 | 20 | 43 | 2.16 | 652 | 28.7 | 212 | 5.1 | 8.6 | 0.14 | 2.3 | 73.2 |
| 10 | 20 | 36 | 1.80 | 499 | 52.9 | 273 | 3.6 | 6.5 | 0.19 | 2.5 | 45.6 |
| 11 | 30 | 66 | 2.22 | 1267 | 28.4 | 156 | 3.8 | 6.4 | 0.18 | 1.8 | 144.9 |
| 12 | 30 | 52 | 1.75 | 1104 | 27.8 | 172 | 4.3 | 6.9 | 0.16 | 1.5 | 145.6 |
| 13 | 30 | 53 | 1.70 | 1286 | 24.2 | 265 | 7.6 | 11.9 | 0.09 | 2.2 | 97.7 |
| Mean | | | | | 53.7 | 416 | 5.2 | 8.6 | 0.15 | 2.1 | |
| SD | | | | | 25.5 | 326 | 2.2 | 3.2 | 0.04 | 0.6 | |

**Table 5A** Intraperitoneal pharmacokinetics of total topotecan (Please note the difference × 1000 in units, mg versus μg as in Table 4A and B)

| Patient no. | Dose (mg/m²) | Absolute dose (mg) | Body surface (m²) | AUC 0 → ∞ (h/mg/L) | CL (L/h/m²) | $V_{ss}$ (L/m²) | T1/2 (h) | MRT (h) | $k_{el}$ (x/h) | $C_{max}$ (mg/L) |
|---|---|---|---|---|---|---|---|---|---|---|
| 3 | 10 | 16 | 1.60 | 30 | 0.3 | 1.3 | 1.8 | 2.6 | 0.38 | 5.72 |
| 5 | 15 | 28 | 1.78 | 76 | 0.2 | 3.7 | 6.6 | 9.5 | 0.11 | 8.93 |
| 6 | 15 | 28 | 1.80 | 23 | 0.9 | 4.1 | 1.8 | 2.6 | 0.38 | 9.11 |
| 7 | 15 | 28 | 1.80 | 20 | 0.9 | 4.4 | 1.8 | 2.6 | 0.39 | 7.36 |
| 8 | 20 | 35 | 1.75 | 162 | 0.2 | 0.9 | 2.3 | 3.3 | 0.30 | 36.15 |
| 9 | 20 | 43 | 2.16 | 121 | 0.4 | 2.7 | 2.2 | 3.2 | 0.31 | 11.30 |
| 11 | 30 | 66 | 2.22 | 133 | 0.3 | 2.0 | 2.3 | 3.4 | 0.30 | 34.40 |
| Mean | | | | | 0.4 | 2.7 | 2.7 | 3.9 | 0.31 | |
| SD | | | | | 0.3 | 1.4 | 1.7 | 2.5 | 0.10 | |

**Table 5B** Intraperitoneal pharmacokinetics of lactone (Please note the difference × 1000 in units, μg versus mg in Table 4A and B)

| Patient no. | Dose (mg/m²) | Absolute dose (mg) | Body surface (m²) | AUC 0 → ∞ (h/mg/L) | CL (L/h/m²) | $V_{ss}$ (L/m²) | T1/2 (h) | MRT (h) | $k_{el}$ (x/h) | $C_{max}$ (mg/L) |
|---|---|---|---|---|---|---|---|---|---|---|
| 8 | 20 | 35 | 1.75 | 85 | 0.2 | 2.8 | 4.6 | 6.7 | 0.15 | 12.1 |
| 9 | 20 | 43 | 2.16 | 18 | 1.2 | 3.6 | 1.0 | 1.4 | 0.71 | 10.5 |
| 11 | 30 | 66 | 2.22 | 39 | 1.0 | 4.2 | 1.3 | 1.8 | 0.55 | 21.5 |
| Mean | | | | | 0.8 | 3.5 | 2.3 | 3.3 | 0.47 | |
| SD | | | | | 0.5 | 0.7 | 2.0 | 2.9 | 0.29 | |

 © 2001 Cancer Research Campaign

(Figure 2) and R = 0.91 $P < 0.01$ for lactone (Figure 3). The mean plasma clearance of total topotecan was 13.4 ± 2.5 L/h/m² with a mean volume of distribution ($V_{ss}$) of 69.9 ± 25.4 L/m². The mean plasma clearance of lactone was 53.7 ± 25.5 L/h/m² with a mean volume of distribution ($V_{ss}$) of 416 ± 326 L/m². It might be better to speak of 'apparent' plasma clearance (CI/F) and volume of distribution ($V_{ss}$/F), as the exact fraction (of the total amount of active lactone administered IP) which reaches the plasma compartment is not known. The AUC ratio between lactone and total topotecan in plasma is shown in Figure 4, illustrating a proportional relationship with the dose, R = 0.84, $P < 0.01$.

Figure 5 shows the peritoneal and plasma concentration versus time curve of total topotecan and lactone for one representative patient at 30 mg/m², indicating that peritoneal concentrations are about 100 times higher than plasma levels and that plasma topotecan levels were sustained during several hours above a threshold concentration of 100 µg/L, which is reported as an active concentration in vitro (Burris et al, 1992).

### DISCUSSION

The activity of single-agent topotecan in refractory ovarian cancer, and the fact that IP instillation can avoid myelotoxicity, served as the rationale for this study (Markman et al, 1992; Ten Bokkel Huinink et al, 1997). Clinical and pharmacological data of intra-peritoneal topotecan administration are sparse (Pratesi et al, 1995; Plaxe et al, 1998) and topotecan efficacy and pharmacokinetics is mostly studied during IV schedules (Herben et al, 1996; Hoskins et al, 1998). The recommended cumulative dose of topotecan in these studies lies between 4 and 22.5 mg/m²: 4 mg/m² as 24-h continuous IP infusion (Plaxe et al, 1998), 7.5 mg/m² as daily × 5 bolus IV (Ten Bokkel Huinink et al, 1997), 8–10 mg/m² as a 24-h continuous IV infusion (Abbruzzese et al, 1993; Van Warmerdam et al, 1995), 10.5 mg/m² as a continuous 5-day infusion (Kantarjian et al, 1993; Rowinsky et al, 1994) or 12.6–16.8 mg/m² as a continuous 21-day IV infusion (Hochster et al, 1995) and 17.5–22.5 mg/m² as daily × 5 IV bolus combined with G-CSF (Rowinsky et al, 1992; Wall et al, 1992; Rowinsky et al, 1996). Repeated exposure seems to be more active, as in the clinic a 5-day schedule has been proven to be more effective than a continuous infusion over 24 h (Hoskins et al, 1998), in a so-called 'pick the winner' comparative phase II study design.

The acute and severely toxic event in the third patient on a dose of 30 mg/m² was considered to be dose-limiting and a dose of 20 mg/m² topotecan was thought to constitute the maximum tolerated dose (MTD) of topotecan. However, up to this point other WHO grade haematological and non-haematological toxicity was considered acceptable. If this acute toxic event in one

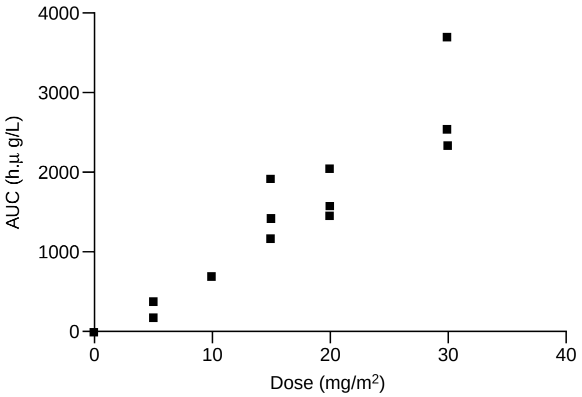

**Figure 2** AUC of plasma total topotecan versus dose administered in µg.h/L for individual patients, R = 0.95. Please note the difference in units (:1000) versus the peritoneal drug levels in Figure 1

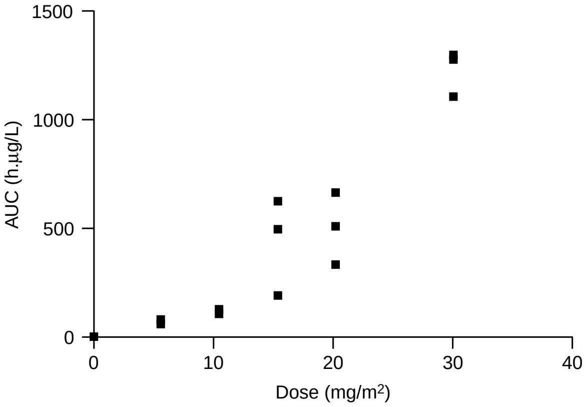

**Figure 3** AUC of plasma lactone topotecan versus dose administered in µg.h/L for individual patients, R = 0.91. Please note the difference in units (:1000) versus the peritoneal drug levels in Figure 1

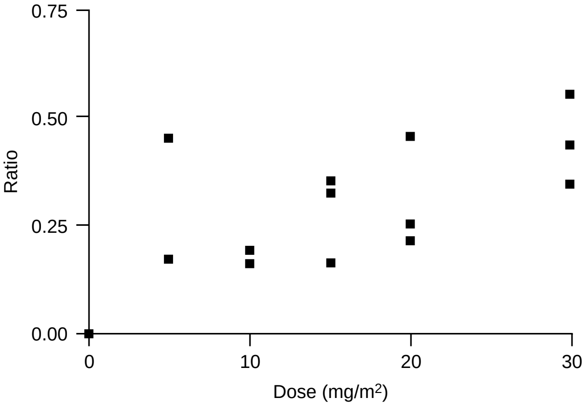

**Figure 4** Ratio between plasma AUC of lactone and total topotecan for individual patients, R = 0.84

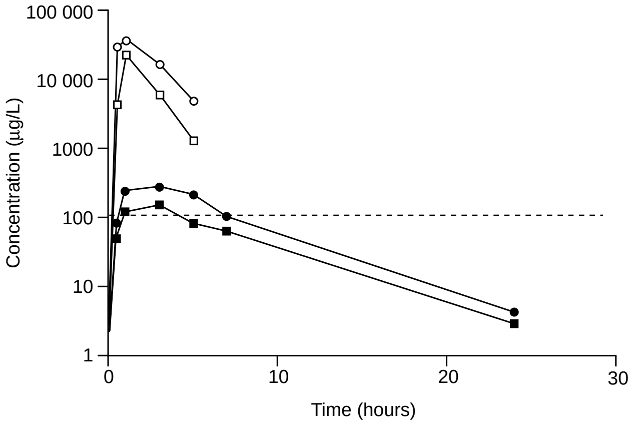

**Figure 5** Plasma and IP concentration of lactone and total topotecan in peritoneal fluid and plasma in a representative patient after 30 mg/m² topotecan IP. The squares represent lactone, the circles total topotecan; open symbols are peritoneal samples, closed symbols are plasma samples. The interrupted line represents a minimal inhibitory topotecan concentration in vitro of 100 µg/L

© 2001 Cancer Research Campaign patient would be considered as a random phenomenon, even a dose of 30 mg/m$^2$ might be acceptable, as it was well-tolerated otherwise.

In vitro, cytotoxic activity of topotecan has been demonstrated for continuous exposure above a threshold concentration of 100 µg/L (Burris et al, 1992). Other studies mention cytotoxic concentrations of 5.5 µg/L (Mi et al, 1995), 13.7–229 µg/L (Pizao et al, 1994) and 128 µg/L (Tanizawa et al, 1994), depending on the methods used in the assay. In this study, plasma topotecan concentrations required for cytotoxicity in vitro were reached from the 15 mg/m$^2$ dose level onwards. In the peritoneal samples, a limit of 100 µg/L is exceeded by a $C_{max}$ of 5000 = a factor 50 at the 10 mg/m$^2$ dose level.

Another observation in this study is that the lactone/total topotecan ratio increases and becomes more favourable at the higher dose levels. As the lactone form is mainly responsible for the cytotoxic effects of topotecan, this implies that at higher dose levels there is not only an absolute but also a relative increase in active topotecan; however, the therapeutic relevance of this finding remains to be established.

The pharmacokinetic data we found after IP administration is in good agreement with the data of others. During continuous IP infusion, Plaxe et al found a terminal T1/2 for peritoneal topotecan of 2.7 ± 0.4 h and for plasma total topotecan a T1/2 of 3.9 ± 2.1 h (Plaxe et al, 1998), which is similar to our data. After IV administration of much lower doses (1.5 mg/m$^2$ bolus), Herben et al reported similar T1/2 for total topotecan, but shorter T1/2 values for the lactone, 2.1 vs 5.5 hours in this study (Rowinsky et al, 1992; Verweij et al, 1993). This can probably be explained because there is a steady flow of lactone from the peritoneal cavity into the plasma compartment, keeping the lactone concentration up, in spite of its simultaneous conversion within the plasma to the less active carboxy compound. As a consequence thereof, Herben et al reported a $V_{ss}$ of 94 L/m$^2$ for lactone, while we found 416 L/m$^2$ for these higher dosages. Therefore, for plasma kinetics, we should probably use the terms 'apparent clearance' (CI/F) and volume of distribution ($V_{ss}$/F), as the exact fraction of the total amount of active lactone administered, which contributes to the concentration of total topotecan and lactone in the plasma, is presently unknown.

The data are in good agreement with those of other studies (Herben et al, 1999; Abbruzzese et al, 1993). In view of a plasma T1/2 for total topotecan of about 2 h, after 12 h the concentration of total topotecan would lie around 1.5% of the initial concentration (6 × T1/2). The concentration/ time curves found after 12 hours will then be mainly determined by influx from the peritoneal stores.

### Toxicity

In most studies, the dose limiting toxicity for topotecan is neutropenia, which is associated with thrombocytopenia in more prolonged schedules (Abbruzzese et al, 1993; Kantarjian et al, 1993; Rowinsky et al, 1994; Van Warmerdam et al, 1995; Hochster et al, 1995). In our study the dose-limiting toxicity was an acute anaphylactoid reaction at the 30 mg/m$^2$ level with only mild myelosuppression and non-haematological toxicity even at this dose. The severity of the toxic reaction precluded a further extension of the number of patients on this dose level. Furthermore, we felt that an active dose has been reached to use in combined treatment in a follow-up study, as the present level already constituted 2–3 times the cumulative dose level of 8–10 mg/m$^2$ (Abruzzesse, 1993; van Warmerdam, 1995) in most IV studies. Further increase of the dose was therefore not deemed clinically meaningful. In sharp contrast with the only other study addressing the intraperitoneal administration of topotecan we arrived at a recommended dosage of 20 mg/m$^2$, instead of 3 mg/m$^2$ when given as a continuous 24-h IP infusion as recommended by the group of Howell et al (Plaxe et al, 1998). This difference can only be explained by the regimen, e.g. continuous infusion, but the exact cause for this difference still remains obscure. It might be that less drug is converted to the open-ring carboxy form, if the drug is given by continuous infusion IP, but this has not been further elucidated by pharmacokinetic studies. Although a lower dose will certainly result in lower cost, little can be said about its efficacy in relation to bolus IP infusion. The data of Hoskins et al were not yet available at the time this study was started, as this favoured a five times daily schedule (Hoskins, 1998).

The need for a more effective first-line regimen in advanced ovarian cancer remains paramount. With the introduction of new classes of chemotherapeutic agents that have demonstrated activity in ovarian cancer, such as topotecan, the question of the optimal first-line regimen remains open (Cannistra, 1999). The renewed interest in multi-agent chemotherapy in ovarian cancer has resulted in trials with combination of cytotoxic drugs given simultaneously, sequentially or as alternating doublets (Herben et al, 1999; Frasci et al, 1999; Cannistra, 1999). However, the limitations of this approach have become apparent; even with growth factor support achieving optimal dosages for the individual agents. Therefore, the key to this problem may lie in IP therapy. IP topotecan can be administered in relevant dosages without major systemic effects. Subsequent trials with multi-agent chemotherapy in ovarian cancer could then employ regimens combining IP topotecan with IV paclitaxel and a platinum compound. This might awaken a renewed interest in the role of IP chemotherapy.

### REFERENCES

Abbruzzese JL, Madden T, Schmidt S, Eaton G and Raber MN (1993) Phase I trial of topotecan (TT) administered by 24-hour infusion without and with G-CSF. *Proc Am Assoc Cancer Res* **34**: 329

Alberts DS, Liu PY, Hannigan EV, O'Toole R, Williams SD, Young JA, Franklin EW, Clarke-Pearson DL, Malviya VK and DuBeshter B (1996) Intraperitoneal cisplatin plus intravenous cyclophosphamide versus intravenous cisplatin plus intravenous cyclophosphamide for stage III ovarian cancer. *N Engl J Med* **335**: 1950–1955

Ardizzonni A, Hansen H, Dombernowsky P, Gamucci T, Kaplan S, Postmus P, Giaccone G, Schaefer B, Wanders J and Verweij J (1997) Topotecan, a new active drug in the second-line treatment of small cell lung cancer: A phase II study in patients with refractory and sensitive disease. *J Clin Oncol* **15**: 2090–2096

Arts HJG, Willemse PHB, Tinga DJ, De Vries EGE and Van der Zee AGJ (1998) Laparoscopic placement of PAP catheters for intraperitoneal chemotherapy in ovarian carcinoma. *Gynecol Oncol* **69**: 32–35

Burris HA, Hanauske AR, Johnson RK, Marshall MH, Kuhn JG, Hilsenbeck SG and Von Hoff DD (1992) Activity of topotecan, a new topo-isomerase I inhibitor, against human tumor colony forming units in vitro. *J Natl Cancer Inst* **84**: 1816–1820

Cannistra SA (1999) Back to the future: Multiagent chemotherapy in ovarian cancer revisited. *J Clin Oncol* **17**: 741–743

Carmichael J and Ozols RF (1996) Topotecan, an attractive new antineoplastic agent: review and current status. *Exp Opin Invest Drugs* **6**: 593–608

Frasci G, Panza N, Comella P, Carteni G, Guida T, Nicolella GP, Natale M, Lombardi R, Apicella A, Pacilio C, Gravina A, Lapenta L and Comella G (1999) Cisplatin-topotecan-paclitaxel weekly administration with G-CSF support for ovarian and small-cell lung cancer patients: a dose-finding study. *Ann Oncol* **10**: 355–358

Friedlander M, Millward MJ, Bell D, Bugat R, Harnett P, Moreno JA, Canpbell L, Varette C, Ripoche V and Kayitalire L (1998) A phase II study of gemcitabine

© 2001 Cancer Research Campaign in platinum pre-treated patients with advanced epithelial ovarian cancer. *Ann Oncol* **9**: 1343–1345

Herben VM, Ten Bokkel Huinink WW and Beijnen JH (1996) Clinical pharmacokinetics of topotecan. *Clin Pharmacokinet* **2**: 85–102

Herben VM, Panday VR, Richel DJ, Schellens JH, Van der Vange N, Rosing H, Beusenberg FD, Hearn S, Doyle E, Beijnen JH and Ten Bokkel Huinink WW (1999) Phase I and pharmacologic study of the combination of paclitaxel, cisplatin and topotecan administered intravenously every 21 days as first-line therapy in patients with advanced ovarian cancer. *J Clin Oncol* **17**: 747–755

Hertzberg RP, Caranfa MJ and Hecht SM (1989) On the mechanism of topoisomerase I inhibition by camptothecin: Evidence for binding to an enzyme-DNA complex. *Biochemistry* **28**: 4629–4638

Hochster H, Liebes L, Zeleniuch-Jacquotte A, Speyer J, Oratz R, Wernz J, Sorich J, Taubes B, Vinci R, Kim A, Fry D, Blum R and Potmesil M (1995) Progressive cleavable complex (CC) formation with 21 day topotecan infusion: A phase I pharmacodynamic study. *Proc Am Soc Clin Oncol* **14**: 1496

Hofstra LS, De Vries EGE, Mulder NH and Willemse PHB (2000) Intraperitoneal chemotherapy in ovarian cancer. *Cancer Treat Rev* **26**: 133–143

Hoskins P, Eisenhauer E, Beare S, Roy M, Drouin P, Stuart G, Bryson P, Grimshaw R, Capstick V and Zee B (1998) Randomized phase II study of two schedules of topotecan in previously treated patients with ovarian cancer: a National Cancer Institute of Canada Clinical Trials Group study. *J Clin Oncol* **16**: 2233–2237

Hsiang YH, Lihou MG and Liu LF (1989) Arrest of replicating forks by drug-stabilized topoisomere I-DNA cleavable complexes as a mechanism of cell killing by camptothecin. *Cancer Res* **49**: 5077–5082

Kantarjian HM, Beran M, Ellis A, Zwelling L, O'Brien S, Cazenave L, Koller C, Rios MB, Plunkett W and Keating MJ (1993) Phase I study of topotecan: a new topoisomerase I inhibitor, in patients with refractory or relapsed acute leukemia. *Blood* **81**: 1146–1151

Kingsbury WD, Boehm JC, Jakas DR, Holden KG, Hecht SM, Gallagher G, Caranfa MJ, McCabe FL, Faucette LF and Johnson RK (1991) Synthesis of water-soluble (amino-alkyl)camptothecin analogues: Inhibition of topoisomerase I and antitumor activity. *J Med Chem* **34**: 98–107

Kudelka AP, Tresukosol D, Edwards CL, Freedman RS, Levenback C, Chantarawiroj P, Gonzalez de Leon C, Kim EE, Madden T, Wallin B, Hord M, Verschraegen C, Raber M and Kavanagh JJ (1996) Phase II study of intravenous topotecan as a 5-day infusion for refractory epithelial ovarian carcinoma. *J Clin Oncol* **14**: 1552–1557

Levenback C, Curtin J, Johnston D, Rubin SC, Yeh S and Hoskins W (1994) Image analysis of the distribution of intraperitoneally administered fluids in patients with ovarian cancer. *Eur J Gynaecol Oncol* **15**: 345–351

Markman M, Rowinsky E, Hakes T, Reichman B, Jones W, Lewis JL Jr, Rubin S, Curtin J, Barakat R and Phillips M (1992) Phase I trial of intraperitoneal taxol: A Gynecologic Oncology Group Study. *J Clin Oncol* **10**: 1485–1491

Markman M (1998a) Intraperitoneal therapy of ovarian cancer. *Semin Oncol* **25**: 356–360

Markman M, Bundy B, Benda J, Alberts D, Wadler S, Fowler J and Carson LF (1998b) Randomized phase III study of intravenous cisplatin, paclitaxel versus moderately high dose IV carboplatin followed by paclitaxel and intraperitoneal cisplatin in optimal residual ovarian cancer. An Intergroup Trial (SWOG, ECOG). *Proc Am Soc Clin Oncol* **17**: 1392

Mi Z, Malak H and Burke TG (1995) Reduced albumin binding promotes the stability and activity of topotecan in human blood. *Biochemistry* **34**: 13722–13728

Neijt JP, Ten Bokkel Huinink WW, Van der Burg ME, Van Oosterom AT, Willemse PHB, Vermorken JB, Van Lindert AC, Heintz AP, Aartsen E, Van Lent M, Trimbos JB and De Meijer AJ (1991) Long-term survival in ovarian cancer: mature data from the Netherlands Joint Study Group for Ovarian Cancer. *Eur J Cancer* **27**: 1367–1372

Pizao P, Smitskamp-Wilms E, Van Ark-Otte J, Beijnen JH, Peters GJ, Pinedo HM and Giaccone G (1994) Anti-proliferative activity of the topoisomerase I inhibitors topotecan and camptotecin, on sub-and postconfluent tumor cell cultures. *Biochem Pharmacol* **48**: 1145–1154

Plaxe SC, Christen RD, O'Quigley J, Braly PS, Freddo JL, McClay E, Heath D and Howell SB (1998) Phase I and pharmacokinetic study of intraperitoneal topotecan. *Invest New Drugs* **16**: 147–153

Pratesi G, Tortoreto M, Corti C, Giardini R and Zunino F (1995) Successful local regional therapy with topotecan of intraperitoneally growing human ovarian carcinoma xenografts. *Br J Cancer* **71**: 525–528

Proost JH and Meijer DK (1992) MW/Pharm, an integrated software package for drug dosage regimen calculation and therapeutic drug monitoring. *Comput Biol Med* **22**: 155–165

Rosing H, Doyle E, Davies BE and Beijnen JH (1995) High-performance liquid chromatographic analysis of the novel new antitumour drug topotecan in human plasma. *J Chromatogr B* **668**: 107–115

Rowinsky EK, Grochow LB, Hendricks CB, Ettinger DS, Forastiere AA, Hurowitz LA, McGuire WP, Sartorius SE, Lubejko BG and Kaufmann SH (1992) Phase I and pharmacologic study of topotecan: A novel topoisomerase I inhibitor. *J Clin Oncol* **10**: 647–656

Rowinsky EK, Adjei A, Donehower RC, Gore SD, Jones RJ, Burke PJ, Cheng YC, Grochow LB and Kaufmann SH (1994) Phase I and pharmacodynamic study of the topoisomerase I inhibitor topotecan in patients with refractory acute leukemia. *J Clin Oncol* **12**: 2193–2203

Rowinsky E, Kaufman S, Baker S, Donehower RC, Gore SD and Burke PJ (1996) Phase I, pharmacologic and pharmacodynamic study of the topoisomerase I inhibitor topotecan (TPT) in adults with refractory acute leukaemia. *Ann Oncol* **7(Suppl)**: 132

Tanizawa A, Fujimori A, Fugimori Y and Pommier Y (1994) Comparison of topoisomerase I inhibition, DNA damage and cytotoxicity of camptotecin derivatives presently in clinical trials. *J Natl Cancer Inst* **86**: 836–842

Ten Bokkel Huinink W, Gore M, Carmichael J, Gordon A, Malfetano J, Hudson I, Broom C, Scarabelli C, Davidson N, Spanczynski M, Bolis G, Malmstrom H, Coleman R, Fields SC and Heron JF (1997) Topotecan versus paclitaxel for the treatment of recurrent epithelial ovarian cancer. *J Clin Oncol* **15**: 2183–2193

Van Warmerdam LJC, Ten Bokkel Huinink WW, Rodenhuis S, Koier I, Davies BE, Rosing H, Maes RA and Beijnen JH (1995) Phase I clinical and pharmacokinetic study of topotecan administered by continuous iv infusion. *J Clin Oncol* **13**: 1768–1776

Verweij J, Lund B, Beijnen J, Planting A, De Boer-Dennert M, Koier I, Rosing H and Hansen H (1993) Phase I and pharmacokinetics study of topotecan, a new topoisomerase I inhibitor. *Ann Oncol* **4**: 673–678

Wall JG, Burris HA, Von Hoff DD, Rodriguez G, Kneuper-Hall R, Shaffer D, O'Rourke T, Brown T, Weiss G and Clark G (1992) A phase I clinical and pharmacokinetic study of the topoisomerase I inhibitor topotecan (SK&F 104864) given as an intravenous bolus every 21 days. *Anticancer Drugs* **3**: 337–345

*© 2001 Cancer Research Campaign*
